# Gout and subsequent increased risk of cardiovascular mortality in non-diabetics aged 50 and above: a population-based cohort study in Taiwan

**DOI:** 10.1186/1471-2261-12-108

**Published:** 2012-11-21

**Authors:** Victor C Kok, Jorng-Tzong Horng, Hsin-Li Lin, Yu-Ching Chen, Yan-Jun Chen, Kuang Fu Cheng

**Affiliations:** 1 Department of Biomedical Informatics, School of Computer Science, Asia University, Taichung, Taiwan; 2 Department of Internal Medicine, Kuang Tien General Hospital, Taichung, Taiwan; 3 Department of Computer Science and Information Engineering, National Central University, Jhongli City, Taiwan; 4 Department of Computer Science and Information Engineering, Asia University, Taichung, Taiwan; 5 Biostatistics Center and Department of Public Health, China Medical University, Taichung, Taiwan

## Abstract

**Background:**

Limited data are available on the risk ratios for fatal cardiovascular disease (CVD) outcome from gout and chronic kidney disease (CKD) in non-diabetic individuals.

**Methods:**

Nationwide population-based retrospective prospective study with a 5-year follow-up to investigate the association between physician-diagnosed gout and CKD in non-diabetics aged 50 and above who had no pre-existing serious CVD and the subsequent risk of death from CVD. Hazard ratios (HR) of CVD mortality were adjusted for gender, age, smoking- and alcoholism-related diagnoses, hypertension, hyperlipidemia, atrial fibrillation and Charlson’s comorbidity index score.

**Results:**

A case cohort (n=164,463) having gout and a control cohort (n= 3,694,377) having no gout were formed. The prevalence of gout in this study was 4.26% whereas that of gout plus CKD was 8.17%. Male to female ratio among the individuals with gout was 3.2:1. The relative risk (RR) of subsequent cardiovascular mortality between the case and control cohort was 1.71 (95% confidence interval (CI), 1.66-1.75). The presence of CKD in nondiabetic subjects with no gout (control group) has a RR of CVD mortality at 3.05 (95% CI, 2.94-3.15). The presence of gout has protective effect on subjects with CKD with a RR of 1.84 (95% CI, 1.71-1.98). As compared with individuals with no gout, the adjusted HR (aHR) for CVD mortality among the individuals with gout was 1.10 (95% CI 1.07-1.13). In a Cox model, when compared with subjects having neither gout nor CKD, the aHR in subjects with no gout but with CKD is 1.76 (95% CI, 1.70-1.82); in subjects with gout but without CKD, 1.10 (1.07-1.13); interestingly, the aHR is attenuated in subjects with concomitant gout plus CKD which is 1.38 (1.29-1.48).

**Conclusions:**

Among non-diabetic individuals aged 50 years or above who had no preceding serious CVD, those with gout were 1.1 times more likely to die from CVD as were individuals without gout. The presence of gout appears to attenuate the risk of subsequent CV mortality in subjects with CKD. Further studies should focus on finding an explanation for the protective effect of gout on CV mortality in patients with CKD.

## Background

Gout is commonly seen in clinics and its prevalence has increased in recent years. It is estimated that approximately 6.1 million adults in the United States have had been diagnosed with gout [1]. Gout or hyperuricemia has long been suspected to increase the risk of cardiovascular disease [2-5]. A recent report from Taiwan using data from a single institute obtained via a health screening program demonstrated that the adjusted hazard ratio of cardiovascular mortality was 1.97 (95% CI 1.08, 3.59) for subjects with gout as compared with subjects with normouricemia [3].

Death from cardiovascular disease (CVD) encompassed coronary heart disease (CHD), which was manifested by myocardial infarction, angina pectoris, heart failure, and coronary death; cerebrovascular disease as manifested by stroke and/or transient ischemic attack; peripheral artery disease and aortic atherosclerosis with thoracic or abdominal aortic aneurysm. It is noted that cardiovascular events in the Asian-Pacific countries consist of relatively fewer from coronary heart disease (CHD) but relatively more from stroke events [6].

Type 2 diabetes has been considered by some as having a similar negative effect on coronary heart disease (CHD) [7-10]. In a population-based 18-year follow-up study of Finnish subjects, type 2 diabetes without prior myocardial infarction and prior myocardial infarction without diabetes resulted in a similar risk of CHD mortality among both men and women. When less stringent criteria for prior history of CHD were used (myocardial infarction or ischemic ECG changes or angina pectoris), type 2 diabetes carried a larger risk than having a prior history of CHD, particularly in women [7]. Thus when diabetes is present together with gout, diabetes *per se* is a significant confounding factor with respect to the cardiovascular mortality. Based on the current observations as stated above, we therefore have investigated, in the absence of any influence from diabetes, whether individuals with gout carry a higher risk of mortality from cardiovascular cause.

Similar to the observations that diabetes mellitus is a risk factor contributing to CVD mortality, patients with chronic kidney disease also have increased risk of cardiovascular mortality[11-14]. Therefore it is interesting to investigate the association between gout with or without CKD and the subsequent CVD mortality.

We therefore carried out a nationwide population-based study to examine the association between physician-diagnosed gout in non-diabetic subjects aged 50 years or above and with or without concomitant CKD and subsequent CVD mortality.

## Methods

### Database

This study was designed as a population-based retrospective cohort study using the Taiwan National Health Insurance Research Database (NHIRD). Taiwan National Health Insurance (NHI) program started in March 1, 1995. As of 2007, 98.4% of Taiwan’s population (22.96 million individuals) were enrolled in this program. The NHIRD contains a number of large computerized databases that include registration files and original data on claims’ reimbursement that are derived from the insurance system held by the Bureau of National Health Insurance of Taiwan (BNHI); these databases are maintained by the National Health Research Institutes (NHRI) of Taiwan and are provided to researchers for academic research purposes. These data files are de-identified by scrambling the identification codes of both the individuals and medical facilities; information on individuals is then sent to the National Health Research Institutes and forms the original files of NHIRD. This study adhered to strict confidentiality guidelines that are in accordance with the regulations regarding personal electronic data and privacy protection. As the data files consist of unidentified secondary data, the study was exempted from a full review by the Institutional Review Board.

We utilized the databases for admissions and outpatient visits for the study cohorts, both of which include information on individuals’ characteristics, and include sex, date of birth, date of admission, date of discharge, dates of visits, admission diagnoses, and outpatient visit diagnoses.

### Study cohorts

From the year 2003, we pinpointed individuals who were aged 50 and above from the total enrollees available on the databases who had sought medical services from the Taiwan National Health Insurance system. Subjects with incomplete data or null value (5.6% of the total enrollees in 2003) were excluded. Subjects who had suffered from a severe form of cardiovascular disease or diseases during the preceding year were also excluded. The severe cardiovascular diseases included malignant hypertension, hypertensive nephropathy, myocardial infarction or any form of congestive heart failure as well as having had coronary artery bypass surgery or percutaneous transluminal coronary angioplasty. Additionally, intracerebral hemorrhage, intracranial hemorrhage, occlusion/stenosis of the precerebral arteries and occlusion of cerebral arteries were also excluded (see Appendix: Table 4). By excluding these individuals, we ensured that the control group and case group did not have existing severe comorbidities such as ischemic heart disease, an old cerebrovascular accident and carotid arterial stenosis. Finally, we excluded every remaining subject that also had either type 1 or type 2 diabetes.

Using the diagnostic variables in admission and outpatient data files from the NHI database, we sorted the study case subjects into those with gout-related diagnoses, i.e., showing codes under 274 of the International Classification of Disease, 9th Revision, Clinical Modification (ICD-9-CM) (see Appendix: Table 5)[15] and those without such a diagnosis. Under ICD-9-CM code 274, individuals with gouty arthropathy (274.0), gouty nephropathy (274.1), gouty iritis (274.89), gouty tophi (274.8), and uric acid nephrolithiasis (274.11) were included. Validation of these physician-diagnosed gout was ensured by verification that there were at least three separate outpatient visits by the same individual. We use gout and gout-related diagnoses interchangeably in this paper.

Demographic data such as age, gender, smoking-related diagnosis, alcoholism-related diagnosis, and Charlson’s comorbidity index (CCI) were collected for further adjustment. Any comorbid medical conditions were identified using their standard ICD-9-CM codes and were used to calculate cumulatively the established Charlson-Deyo comorbidity index for each individual. The Charlson-Deyo comorbidity index score, adapted from the Charlson index for use with ICD-9-CM coded administrative databases, contains 17 weighted categories related to chronic concomitant diseases and is able to predict the subsequent 1-year mortality among inpatients. Each category has a score between 1 and 6 points (1 point for myocardial infarction, congestive heart failure, peripheral vascular disease, cerebrovascular disease, dementia, chronic pulmonary disease, rheumatological disease, peptic ulcer disease, mild liver disease, and diabetes without organ damage; 2 points for diabetes with organ damage, hemiplegia or paraplegia, severe renal disease, any malignancy including leukemia and lymphoma; 3 points for severe liver disease; 6 points for metastatic solid tumor and HIV infection), and sum of these scores is regarded as a measure of the burden of comorbidity[16,17]. To avoid double-counting and possible over-adjustment in a regression model, chronic kidney disease was excluded from the CCI score.

After the stringent selection process of the subject individuals as stated above, ultimately the study cases were non-diabetic individuals with gout, aged 50 and above, who had no severe hypertensive, cerebrovascular or cardiovascular disease. In order to elucidate the impact of chronic kidney disease on the analyses, both case and control groups were dichotomized into two groups by the status of CKD. Chronic kidney diseases are defined based on ICD-9-cm codes given for at least three times in the reimbursement data. Cases were then followed up until year 2007. The risk for CVD mortality associated with gout was evaluated using relative risk (RR) calculation and hazard ratio (HR) in Cox proportional hazards regression.

The control group in this study consists of those without any gout-related diagnosis after having gone through the same aforementioned selection process.

### Survival data

We checked the National Death Registry maintained by the Department of Health of Taiwan for death from cardiovascular disease and well as to confirm the survival of each individual case within the two cohort populations included that make up our study. The status and causes of death were ascertained from the Taiwan National Death Registry 2003–2007.

### Statistical analysis

SAS statistical package (SAS System for Windows, version 8.2, SAS Institute, Cary, NC, USA) was used for pre-analysis data file merging and other data management before statistical analysis. Statistical analysis was performed using SPSSsoftware (version 17.0, SPSS Inc., Chicago, Illinois, USA). All statistical tests were two-sided. Values of *p* < 0.05 were considered statistically significant. The risk of death due to CVD that was associated with gout was evaluated using Cox proportional hazards analysis. All Cox regression models included the following covariates: gender, age, smoking-related diagnosis as a surrogate for cigarette smoking history and alcoholism-related diagnoses as a surrogate for chronic alcoholism history, hypertension, hyperlipidemia, atrial fibrillation and Charlson’s co-morbidity index (CCI) score. To avoid double-counting and thus over-adjustment during multivariable regression model, in subjects with chronic kidney disease, CCI score did not consider chronic renal failure as a comorbidity.

Hazard ratios with a 95% confidence interval were calculated. The relative risks (RR) of CVD mortality for both genders were then calculated and analyzed by Chi-square test (2x2).

## Results

**Figure 1:**
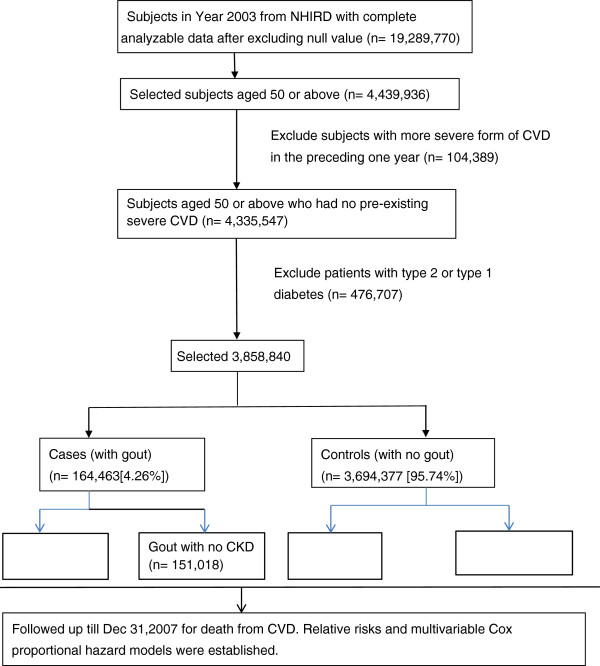
**Flowchart illustrating the selection of cases and controls and follow up.** Abbreviations: CKD, chronic kidney disease; CVD, cardiovascular disease; NHIRD, National Health Insurance Research Database.

Based on the NHIRD, there were 20,439,3939 enrollees during the whole year 2003. After excluding enrollees data containing null value (or missing data) or incomplete data which was approximately 5.6% of the total enrollees’ record, 19,289,770 were retained as the source for study. Out of these, there were 4,439,936 individuals who were aged 50 year-old or older. A total of 104,389 individuals with preexisting severe CVD during the preceding year of this case-cohort study were then excluded. Of the remaining 4,335,547 individuals, 476,707 were excluded next because they were suffering from either type 1 or type 2 diabetes. This left 3,858,840 individuals of which 164,436 (4.26% of the non-diabetic individuals aged 50 or over and without a preexisting excluding condition), had gout. Finally, the individuals without gout forming the control group totaled 3,694,377 (Figure 1). The proportion of CKD in both groups is different, with higher proportion of CKD in the case group, approximately 8.2% versus 1.4% in the control group. There is no causal relationship (gout to CKD or the reverse) suggested by this cross-sectional data.

The male to female ratio among the subjects with gout was calculated as 3.2:1 and consisted of 125,311 men and 39,152 women. The case group as compared with the control group without gout, has higher prevalence of hypertension (27.8% versus 10.1% in the control group), hyperlipidemia (5.8% versus 1.4%), smoking-related diagnosis (10.4% vs. 6.7%), alcoholism-related diagnosis (1.8% vs. 0.7%) and uremia (1.4% vs. 0.5%) (Table 1).

**Table 1:** Patient demographics and disease and treatment characteristics

**Characteristic**	**Overall**	**Case group**	**Control group**	***P*-value**
	**3,858,840**	**164,463 [%]**	**3,694,377 [%]**	
With chronic kidney disease	66,476	13,445 [8.18]	53,031 [1.44]	<.0001
Without chronic kidney disease	3,792,364	151,018	3,641,346	
Age group, years				
50-59	1,750,009	54,586 [33.19]	1,695,423	<.0001
60-69	1,097,797	50,413 [30.65]	1,047,384	
70-79	734,932	45,041 [27.39]	689,891	
≥80	276,102	14,423 [8.77]	261,679	
Sex				
Male	1,863,738	125,311 [76.19]	1,738,427 [47.06]	<.0001
Female	1,995,102	39,152	1,955,950	
Comorbidities				
Hypertension	419,678	45,693 [27.8]	373,985[10.1]	<.0001
Hyperlipidemia	60,744	9,521 [5.8]	51,223 [1.4]	<.0001
Smoking-related diagnosis	262,913	17,183 [10.4]	245,730 [6.7]	<.0001
Alcoholism-related diagnosis	28,305	2,891 [1.8]	25,414 [0.7]	<.0001
Uremia	21,526	2,228 [1.4]	19,298 [0.5]	<.0001
Charlson’s comorbidities Index score**†**				
0	3,099,342	106,542	2,992,800	0.0336
1	494,593	35,179	459,414	
2	178,885	14,215	164,670	
3	50,181	5,264	44,917	
4	16,497	1,858	14,639	
5	5,752	659	5,093	
6	2,360	261	2,099	
7	1,269	101	1,168	
8	6,321	204	6,117	
9	2,384	100	2,284	
10+	1,256	80	1,176	
Follow-up in years				
0	63,220	2,682	60,538	-
1	65,186	4,115	61,071	
2	67,987	4,446	63,541	
3	67,136	4,484	62,652	
4	3,519,426	144,228	3,375,198	
5	75,885	4,508	71,377	

**†**Mann-Whitney U test.

All subjects in case and control groups were followed up to the end of year 2007. A total of 74,367 subjects in the control group and 5,650 individuals in the case group were identified as dying of cardiovascular disease during follow-up. The relative risk of CVD mortality among subjects with gout was calculated to be 1.71 (95% CI = 1.66 – 1.75) (Table 2). In the control group, subjects with chronic kidney disease (CKD) have approximately 3-fold risk of dying from CVD than subjects without CKD (RR = 3.05, 95% CI = 2.94-3.15). However this magnitude of risk is markedly diminished in the case group subjects with CKD, i.e., subjects with both gout and CKD, with RR of 1.84, 95% CI 1.71-1.98 (Table 2).

**Table 2:** Relative risks of dying from CVD by the presence of gout plus/minus CKD

		**Number at risk**	**Number of deaths**	**Relative risk**	**95% CI**
Overall					
	Control	3,694,377	74,367	1 [Reference]	-
	Case	164,463	5,650	1.71	1.66-1.75
Control with or without CKD					
	No CKD	3,641,346	71,209	1 [Reference]	-
	With CKD	53,031	3,158	3.05	2.94-3.15
Case with or without CKD					
	No CKD	151,018	4,853	1 [Reference]	-
	With CKD	13,445	797	1.84	1.71-1.98

*CVD *cardiovascular disease; *CI *confidence interval; Chi-square test (2x2) was used for the RR calculation.

The crude hazard ratio (HR) of CVD mortality among the case group was found to be 1.69 with 95% CI from 1.7 to 1.7 (*p* value <0.0001). After adjustments made for gender, age, smoking-related diagnosis, alcoholism-related diagnosis, hypertension, hyperlipidemia and Charlson’s co-morbidity index, the adjusted HR was 1.10 with 95% CI of 1.07 to 1.13 (*p* value <0.0001). This confirms that gout alone in this studied population of nondiabetic subjects aged 50 or above contributes statistically significantly, as a risk factor to subsequent death from CVD cause. In another words, a person in the case group is 1.1 times as likely to die of CVD cause over the next 5 years in comparison with a person in the control group (Table 3, model 1).

**Table 3:** Hazard ratios of subsequent death from CVD (2005.1.1-2007.12.31)

**Cox models**	**Crude HR**	**95%CI**		**SE**	***P*-Value**	Adjusted HR*	**95%CI**		**SE**	***P*-Value**
		**Low**	**Up**				**Low**	**Up**		
Model 1										
Control group (without gout)	1	-	-	-	-	1	-	-	-	-
Case group (with gout)	1.69	1.65	1.74	0.01	<.0001	1.10	1.07	1.13	0.01	<.0001
Model 2										
Gout but no CKD	1	-	-	-	-	1	-	-	-	-
Gout plus CKD	1.99	1.85	2.14	0.04	<.0001	1.40	1.29	1.51	0.04	<.0001
Model 3										
Control without CKD	1	-	-	-	-	1	-	-	-	-
Control with CKD	3.35	3.24	3.47	0.02	<.0001	1.74	1.68	1.81	0.02	<.0001
Model 4										
Control without CKD	1	-	-	-	-	1	-	-	-	-
Control with CKD	3.36	3.24	3.48	0.02	<.0001	1.76	1.70	1.82	0.02	<.0001
Gout but no CKD	1.62	1.58	1.67	0.01	<.0001	1.10	1.07	1.13	0.02	<.0001
Gout plus CKD	3.21	2.99	3.44	0.04	<.0001	1.38	1.29	1.48	0.04	<.0001

*Adjustments were made in these Cox models for gender, age, smoking and alcoholism-related diagnoses, hypertension, hyperlipidemia, atrial fibrillation and Charlson’s co-morbidity index (CCI) score. To avoid double-counting (over-adjustment), in subjects with chronic kidney disease, CCI score did not consider chronic renal failure as a comorbidity.

*CVD *cardiovascular disease; *CI *confidence interval; *CKD* chronic kidney disease. *HR *hazard ratio which was calculated by Cox proportional time-to-event survival analysis; *SE *standard error.

In the case cohort with gout, the presence of CKD has a crude HR of 1.99 of subsequent CVD mortality when compared with subjects with gout but no CKD. The adjusted HR is 1.40 (95% CI, 1.29 – 1.51) (Table 3, model 2).

In subjects without gout, chronic kidney disease (CKD) has higher crude HR of 3.35 (95% CI, 3.24 – 3.47) of subsequent death from CVD in the next 5 years. After adjustments made in the multivariable Cox regression model, the adjusted HR is 1.74 (95% CI, 1.68 – 1.81) (Table 3, model 3).

The interplay from gout and CKD to the subsequent death from CVD can be illustrated from the fourth Cox model in Table 3 where when compared with “Control without CKD” (subjects with neither gout nor CKD), the adjusted HR was 1.76 (95% CI, 1.70 – 1.82) in “control with CKD”; 1.10 (95% CI, 1.07 – 1.13) in cases with gout but without CKD; 1.38 (95% CI, 1.29 - 1.48) in cases with gout plus concomitant CKD. After adjustment was made for gender, age, smoking-related diagnosis, alcoholism-related diagnosis, hypertension, hyperlipidemia, atrial fibrillation and Charlson’s comorbidity index score, the adjusted HR is attenuated from 1.76 (in subjects without gout but with CKD) to 1.38 (in subjects with gout and with CKD) when gout is present concomitantly with CKD.

## Discussion

Cardiovascular disease (CVD) mortality is the leading cause of death in most developed countries. Coronary heart disease (CHD), one of the four diagnostic categories of CVD, accounts for approximately one third to one half of CVD cases. It is well established that diabetes mellitus (DM) and chronic kidney disease (CKD) are considered as two most important CHD risk equivalents [7,12,14,18]. Therefore, when this study is to test whether gout is an emerging risk factor for CVD, the confounding factors from DM and CKD should be tackled first using a different study design.

To our knowledge, this study is the first of its kind to examine the association of gout and CVD mortality using a nationwide population-based cohort study with an *ad hoc* design to deliberately exclude patients with DM and then stratify cohorts into groups with or without CKD at the beginning of the study. The advantage of excluding all patients with type 2 diabetes before forming study cohorts precludes the necessity in the future resorting to statistical adjustments which would be suboptimal in terms of evidence-based approach. This study also excludes patients with pre-existing major cardiovascular and/or cerebrovascular co-morbidities in the preceding one year of the study. The strength of this study includes the complete follow-up with no drop-outs due to nationwide registration of the subjects’ use of any medical resource in the country.

Our results show that the distribution of cases of gout declines gradually from 33.19% in the age group of 50–59 years down to 8.77% in the age group of ≥ 80 years (Table 1). This difference of the prevalence of gout in different age group can be explained by the fact that subjects who live longer are less likely afflicted with gout problems or its related complications. The strengths of this study is using smoking-related diagnosis, alcoholism-related diagnosis, Charlson-Deyo comorbidity index score to avoid the occurrence of recall bias from interview-based or questionnaire-based study design [16,17].

Our results show that gout is an independent risk factor for CVD mortality. The relative risk for CVD mortality as the cause of death in the next five years is 1.71 with a narrow 95% confidence interval of 1.66 to 1.75. The crude hazard ratio for subsequent death from CVD in subjects with gout is 1.69 (95% CI, 1.65-1.74). After adjustment made for gender, age, smoking-related diagnosis, alcoholism-related diagnosis, hypertension, hyperlipidemia, atrial fibrillation and Charlson’s comorbidity index score, the multivariable Cox regression analysis still shows a statistically significant hazard ratio of 1.10 (95% CI, 1.07-1.13). This confirms that gout alone modestly increases the risk for subsequent CVD mortality in the next five years.

Review of the literature on the association between gout and CHD or CVD mortality discloses four relevant studies of heterogeneous study design and different follow-up time [3,5,19,20]. The Health Professionals Follow Up Study and the Multiple Risk Factor Intervention Trial included Caucasian male subjects only[5,19]. The HR for cardiovascular mortality in the latter trial was not statistically significant exhibiting an adjusted HR of 1.21 (95% CI, 0.99-1.49) [19]. A single institution observational study in Taiwan using health screening program subjects shows that the adjusted HR for CVD death is 1.97 (95% CI, 1.08-3.59) in participants with gout [3]. This study had short follow up time (mean, 56 months) and did not make adequate adjustment for significant confounders such as medical comorbidities. A recently published study from Singapore which included only Chinese aged 45 to 74 years with gout has demonstrated an HR for CHD mortality of 1.38 (95% CI, 1.10 - 1.73) [20]. Nevertheless, this study necessitated the participants to recall a history of previously diagnosed gout as their standard definition of gout. Furthermore, the Singaporean study was not able to adequately adjust for the participants’ coexisting medical co-morbidities. Our present study was able to use physician-diagnosed gout as the basis of disease confirmation and adding Charlson comorbidity index score for adjustment in the multivariable Cox regression.

This study shows that gout coexists with CKD in 8.18% in subjects with gout whereas only 1.44% of subjects in the control group have had CKD. No causal relationship can be extrapolated from the current study design. Our study demonstrated that in subjects with no gout, the presence of CKD exhibited a RR of 3.05 (95% CI, 2.94 – 3.15) of CVD mortality. It is surprising to note that gout attenuates the association between CKD and the risk for CVD mortality in patients with CKD coexisting with gout demonstrating a RR of 1.84 (95% CI, 1.71 - 1.98). In a Cox proportional hazard model with multivariable adjustment, when compared to control without CKD (subjects with neither gout nor CKD), the group of subjects with no gout but with CKD has an adjusted HR of 1.76 (95% CI, 1.70 – 1.82), however, the coexistence of gout plus CKD pulls down the adjusted HR to 1.38 (95% CI, 1.29 - 1.48) (Table 3).

Our study is the first to show that the presence of gout may have protective effects against cardiovascular mortality in patients with chronic kidney disease. The possible explanations for this observation may not be the gout per se but on the medications used for managing gout such as urate-lowering drug, allopurinol [21-25]. Allopurinol, a xanthine oxidase inhibitor, is the most commonly used medication for urate-lowering purpose in gout and hyperuricemia. Preclinical data have shown that allopurinol has protective effect on both myocardial and renal ischemia-reperfusion injury [22,24]. In an observation study, allopurinol was associated with a lower risk of all-cause mortality (HR 0.78; 95% CI 0.67, 0.91) in subjects with hyperuricemia [23]. A small-scale prospective open-label randomized trial carried out in patients with chronic kidney disease with patients randomly assigned to treatment with allopurinol 100 mg/day (n = 57) or to continue the usual therapy (n = 56), allopurinol decreases C-reactive protein and slows down the progression of renal disease in patients with CKD [21]. In addition, allopurinol reduces cardiovascular and hospitalization risk in these subjects. Allopurinol treatment reduces risk of cardiovascular events in 71% compared with standard therapy with an adjusted HR for new cardiovascular events such as congestive heart failure, cerebrovascular accidents, ischemic coronary events, and peripheral arteriopathy, at 0.29 (95% CI, 0.09 – 0.86) [21]. Therefore, our present study with complete follow up demonstrating attenuated risks for subsequent cardiovascular mortality by the coexistence of gout and CKD may be explained by allopurinol prescribed in patients with gout among other explanatory variables. Further studies should examine the association between allopurinol use and the cardiovascular mortality.

One of the potential limitations of this study is whether the diagnosis of physician-diagnosed gout possibly contains pseudogout and other crystalline arthritides. Because each gout case was identified as having made three separate visits (same ICD code given on at least three clinical encounters) and this is a strict criterion for physician-diagnosed gout adopted by almost all research on administrative database, the potential misclassification bias is considered very modest due to relative ease of making a gout diagnosis by physicians in Taiwan [26]. We do not have access to the relevant biochemical datasets and lifestyle personal histories such as abdominal obesity, psychosocial factors, consumption of fruits and vegetables, and regular physical activity. This is because individual identities are not available due to the de-identification of the individuals within the NHI databases. Another potential limitation is that data for medication and treatment for gout and CKD and the adherence to treatment were too complex to incorporate in the covariate analyses. Furthermore, the impact of undiagnosed diabetes mellitus cannot be excluded because diabetes and metabolic syndrome is related to both gout and cardiovascular mortality [27-29].

In summary, the results clearly show that, among individuals without diabetes who have no major cardio-cerebrovascular disease, gout presents a higher risk of death from cardiovascular causes.

## Conclusions

Among non-diabetic individuals aged 50 years or above who had no preceding serious CVD, those with gout were 1.1 times more likely to die from cardiovascular disease as were individuals without gout in the next five years. Confounding factors such as the impact of undiagnosed diabetes mellitus cannot be excluded. In conclusion, we suggest that gout although significant is not a powerful risk factor for future cardiovascular mortality as evidenced from this population-based nationwide observation study with a prospective cohort. The protective effect against cardiovascular mortality evidenced by the attenuation of risk in subjects with gout plus chronic kidney disease in this study should be further explored and confirmed in the future studies.

## Appendix

Table 4 The ICD-9-cm codes for severe forms of cardiovascular diseases

**Table 4:** The ICD-9-cm codes for severe forms of cardiovascular diseases

**ICD-9-cm**	**Disease**
402.00	Hypertensive heart disease Malignant Without heart failure
402.01	Hypertensive heart disease Malignant With heart failure
402.11	Hypertensive heart disease Benign With heart failure
402.91	Hypertensive heart disease Unspecified With heart failure
404.00	Hypertensive heart and chronic kidney disease Malignant
404.01	Hypertensive heart and chronic kidney disease Malignant
404.03	Hypertensive heart and chronic kidney disease Malignant
404.13	Hypertensive heart and chronic kidney disease Benign
404.91	Hypertensive heart and chronic kidney disease Unspecified
404.93	Hypertensive heart and chronic kidney disease Unspecified
414.04	Coronary atherosclerosis of artery bypass graft
V45.81	Aortocoronary bypass status
V45.82	Percutaneous transluminal coronary angioplasty status
410	Acute myocardial infarction
411	Other acute and subacute forms of ischemic heart disease
429.79	Certain sequelae of myocardial infarction, not elsewhere classified
398.91	Rheumatic heart failure (congestive)
428.0	Congestive heart failure, unspecified
518.83	Chronic respiratory failure
415.19	Pulmonary embolism and infarction Other
573.4	Other disorders of liver: Hepatic infarction
431	Intracerebral hemorrhage
432.0	Intracranial hemorrhage: nontraumatic epidural hemorrhage
432.9	Other and unspecified intracranial hemorrhage Unspecified intracranial hemorrhage
430	Subarachnoid hemorrhage
433	Occlusion and stenosis of precerebral arteries
434	Occlusion of cerebral arteries
997.02	Iatrogenic cerebrovascular infarction or hemorrhage

*ICD-9-cm* International Classification of Diseases, 9th revision; clinical modification, 6th edition

*ICD-9-cm* International Classification of Diseases, 9th revision; clinical modification, 6th edition

Table 5 Gout and gout-related diagnoses and manifestations

**Table 5:** Gout and gout-related diagnoses and manifestations

**ICD-9-cm**	**Disease**
274	gout
274.0	gouty arthropathy
274.1	gouty nephropathy
274.10	gouty nephropathy, unspecified
274.11	uric acid nephrolithiasis
274.19	other gouty nephropathy
274.8	gout with other manifestations
274.81	gouty tophi of ear
274.82	gouty tophi of heart
274.89	other manifestations
274.9	gout , unspecified

*ICD-9-cm*: International Classification of Diseases, 9th revision; clinical modification, 6th edition

ICD-9-cm: International Classification of Diseases, 9th revision; clinical modification, 6th edition

## Competing interests

The authors declare that they have no competing interests.

## Authors’ contributions

VCK and JH conceived of the study, participated in the study design and acquisition of data, analysis and interpretation of data as well as revised the manuscript and gave the final approval of the version to be published. JH was the author who acquired funding for this study. HL, YCC and YJC participated in the design of the study, analysis and interpretation of data. KC participated in the study design, statistical analysis, analysis and interpretation of data, and revised the manuscript. All authors read and approved the final manuscript.

## Pre-publication history

The pre-publication history for this paper can be accessed here:

http://www.biomedcentral.com/1471-2261/12/108/prepub
